# *Astilbe chinensis* Modulates Platelet Function via Impaired MAPK and PLC*γ*2 Expression

**DOI:** 10.1155/2018/3835021

**Published:** 2018-08-05

**Authors:** Bo-Ra Jeon, Muhammad Irfan, Seung Eun Lee, Jeong Hoon Lee, Man Hee Rhee

**Affiliations:** ^1^Laboratory of Physiology and Cell Signaling, College of Veterinary Medicine, Kyungpook National University, Daegu 41566, Republic of Korea; ^2^Department of Herbal Crop Research, National Institute of Horticultural and Herbal Science (NIHHS), Eumseong 27709, Republic of Korea

## Abstract

**Background:**

Platelets play major role in maintaining hemostasis while hyperactivation of platelets may lead to arterial thrombosis. Natural products and ethnomedicine have been shown to reduce the risk of cardiovascular diseases (CVDs).* Astilbe chinensis* is a perennial herb found in China, Korea, Russia, and Japan, which is also known for its medicinal effects, and has been used in Korean traditional medicine to treat inflammation, cancer, chronic bronchitis, and headache. We hypothesized that given herbal plant exhibits pharmacological activities against CVDs, and we specifically explored their effects on platelet function.

**Methodology:**

Platelet aggregation was evaluated using standard light-transmission aggregometry. Intracellular calcium mobilization was assessed using Fura-2/AM, and granule secretion (ATP release) was measured in a luminometer. Fibrinogen binding to integrin *α*_IIb_*β*_3_ was assessed using flow cytometry. Phosphorylation of mitogen-activated protein kinase (MAPK) signaling molecules and activation of the phosphoinositide 3-kinase (PI3K)/Akt were assessed using western blots, and further, glycoprotein VI (GPVI) signaling components were studied using immunoprecipitation.

**Key Results:**

* A. chinensis* extracts potently and significantly inhibited platelet aggregation, calcium mobilization, granule secretion, and fibrinogen binding to integrin *α*_IIb_*β*_3_. Moreover, it significantly inhibited MAPK phosphorylation and expression of GPVI downstream signaling molecules.

**Conclusion:**

* A. chinensis* extract inhibited platelet aggregation and granule secretion and attenuated GPVI downstream signaling, indicating the potential therapeutic effects of this plant extract on the cardiovascular system and platelet function. We suggest that given plant extract may be a potent candidate to treat platelet-related CVDs and to be used as antiplatelet agent.

## 1. Introduction

Currently, cardiovascular diseases (CVDs) are the main cause of morbidity and mortality in developed countries [1]. There are multiple risk factors, but platelets, being a main etiological factor, play a central role in CVDs. Platelet aggregation is a key step in the development and progression of atherosclerotic plaques, which cause narrowing of the blood vessels that can ultimately lead to stroke and heart attack [2, 3]. Hyperactive platelets contribute to thrombosis and are important mediators of atherogenesis. Moreover, intravascular thrombosis is a factor that causes various CVDs. Pharmacological suppression of platelet function has shown great success in reducing thrombotic events, and a number of clinically approved antiplatelet drugs are available to treat cardiovascular ailments. However, these drugs can have serious complications (such as gastric bleeding) and are ineffective in some patients [4, 5], necessitating the need to develop effective and safer approaches to treat and prevent CVDs. One approach may include the use of natural products, like plant extracts, as antithrombotics and anticoagulants [6]. Currently, ethnomedicine and natural products are gaining interest as remedies for CVDs [7], as a number of dietary and herbal compounds have been shown to reduce the risk of CVDs [8].

*Astilbe chinensis *is a perennial herb found in China, Korea, Japan, and Russia and has been used in Korean traditional medicine as an analgesic and antipyretic [9, 10]. Other traditional uses include anti-inflammatory, hepatoprotective, anticancer, and treatment of chronic bronchitis and headache [11]. However, cardiovascular effects of this herb have yet to be explored. To date, there is no report on the antiplatelet activity of* A. chinensis*. In this study, we evaluated the effects of this plant extract on the cardiovascular system, especially on platelet function, and explored the mechanistic aspects of their antiplatelet activities.

## 2. Materials and Methods

### 2.1. Chemicals and Reagents

Collagen, ADP, and thrombin were purchased from Chrono-log Corp. (Havertown, PA, USA). Fura-2/AM and dimethyl sulfoxide (DMSO) were obtained from Sigma-Aldrich (St. Louis, MO, USA). Fibrinogen Alexa Fluor® 488 conjugate was purchased from Molecular Probes (Eugene, OR, USA), and the ATP assay kit was obtained from Biomedical Research Service Center (Buffalo, NY, USA). Antibodies against phospholipase C*γ*2 (PLC*γ*2), phospho-p44/42 (phospho-extracellular signal-regulated kinase (ERK)), p44/42 (ERK), phospho-p38, p38, MEK, phospho-MEK, stress-activated protein kinase (SAPK)/c-Jun N-terminal protein kinase (JNK), phospho-SAPK/JNK, phospho-phosphoinositide 3-kinase (PI3K), PI3K, phospho-Akt, and Akt were acquired from Cell Signaling Technology (Beverly, MA, USA). Ultrapure water was obtained from J. T. Baker (Phillipsburg, NJ, USA). All chemicals were reagent grade.

### 2.2. Sample Preparation

Aerial part of* A. chinensis* plant was collected in Rural Development Administration (RDA) in Suwon city in 2004. The powder (100 g) of* A. chinensis* was extracted with methanol in accelerated solvent extraction system (Dionex, USA) at 50°C and evaporated in rotary evaporator (N-1000, Eyela, Japan). Finally, extract (17 g) from the powder of* A. chinensis* was obtained. Powder was dissolved in DMSO for further use in experiments. Vehicle concentration was kept at less than 0.1%.

### 2.3. Animals

Male Sprague-Dawley (SD) rats (240–260 g) were purchased from Orient Co. (Seoul, Korea) and were acclimatized for one week before conducting the experiments in a special air conditioned animal room with 12/12 h light/dark cycle at a temperature and humidity of 23 ± 2°C and 50 ± 10%, respectively. All animal-related studies were carried out following the Institutional Animal Care and Use Committee (IACUC) guidelines, and the protocols were approved by the Ethics Committee of the College of Veterinary Medicine, Kyungpook National University, Daegu, Korea.

### 2.4. Platelet Preparation

Blood was collected from rats via heart puncture and transferred to a tube containing the anticoagulant, acid citrate dextrose (ACD) solution. Blood was centrifuged at 170 ×*g* for 7 min to obtain platelet-rich plasma (PRP). The PRP was further centrifuged at 350 ×*g* for 7 min to isolate platelets. The concentration of platelets was adjusted to 3 × 10^8^ cells/mL using Tyrode's buffer without calcium (137 mM NaCl, 12 mM NaHCO_3_, 5.5 mM glucose, 2 mM KCl, 1 mM MgCl_2_, and 1 mM NaHPO_4_, pH 7.4), and these platelets were used for aggregation assays. All platelet preparation procedures were performed at room temperature (23 ± 2°C).

### 2.5. Platelet Aggregation Assay and Scanning Electron Microscopy Analysis

Platelet aggregation was performed using a standard technique, light-transmission aggregometry (Chrono-log Corp., Havertown, PA, USA), as previously described [12]. Briefly, washed platelets were preincubated with various concentrations of* A. chinensis* extract or vehicle for 2 min at 37°C in the presence of 1 mM CaCl_2_, followed by stimulation with the agonist, collagen, ADP, or thrombin. The mixture was incubated for 5 min with continuous stirring.

A field emission scanning electron microscope (SU8220, Hitachi) was used to assess extent of platelet aggregation with ultrastructure image and platelet shape change at the Center for Scientific Instrument, Kyungpook National University, Daegu, Korea. Briefly, following the platelet aggregation assay, scanning electron microscopy was performed on collagen-stimulated platelets pretreated with various concentrations of* A. chinensis* extract: (a) Resting, (b) Vehicle, (c) 25 *μ*g/mL, (d) 50 *μ*g/mL, and (e) 100 *μ*g/mL. The platelet mixture was fixed with 0.5% paraformaldehyde (first fixation) and osmium tetroxide (second fixation), dehydrated with various concentrations of ethanol, freeze-dried, and scanned.

### 2.6. Intracellular Calcium Ion Concentration ([Ca^*2*+^]_i_) Measurements

The [Ca^2+^]_*i*_ was assessed using Fura-2/AM as previously described [13]. Briefly, platelets were preincubated with 5 *μ*M Fura-2/AM for 1 h at 37°C. Following incubation, the platelets were washed and treated with* A. chinensis* extract for 1 min in the presence of 1 mM CaCl_2_ at 37°C, followed by stimulation with collagen for 2 min. Fluorescence was recorded using a spectrofluorometer (F-2500, Hitachi, Japan) and [Ca^2+^]_*i*_ was calculated with Schaeffer and Blaustein's method [14], using the following formula: [Ca^2+^]_*i*_ in cytosol = 224 nM × (*F* − *F*_min⁡_)/(*F*_max⁡_ − *F*), where 224 nM is the dissociation constant of the Fura-2-Ca^2+^ complex and *F*_min⁡_ and *F*_max⁡_ represent the fluorescence intensity levels at very low and very high Ca^2+^ concentrations, respectively.

### 2.7. ATP Release Assay

Washed platelets were preincubated with* A. chinensis* extract in the presence of 1 mM CaCl_2_ for 2 min at 37°C and then were stimulated with collagen for 5 min. The aggregation reaction was terminated and the platelet mixture centrifuged. The supernatant was used to measure ATP secretion with a luminometer (GloMax 20/20, Promega, Madison, WI, USA), using an ATP assay kit (Biomedical Research Service Center).

### 2.8. Flow Cytometry

The fibrinogen binding to integrin *α*_IIb_*β*_3_ was quantified using flow cytometry as previously described [15]. Briefly, washed platelets were preincubated with* A. chinensis* extract for 2 min in the presence of 0.1 mM CaCl_2_, followed by stimulation with collagen for 5 min. The stimulated platelets were treated with fibrinogen Alexa Fluor® 488 conjugate (20 *μ*g/mL) for 5 min at room temperature (23 ± 2°C) and were then fixed with 0.5% paraformaldehyde for 30 min at 4°C. Following fixation, platelets were washed thrice in phosphate-buffered saline (PBS). Fluorescence was recorded with a BD FACSCalibur™ flow cytometer (BD Biosciences, San Jose, CA, USA), and data were analyzed using CellQuest™ software (Becton Dickinson Immunocytometry Systems, San Jose, CA, USA).

### 2.9. Immunoprecipitation

Platelets were suspended at 8 × 10^8^ cells/mL in modified Tyrode's-HEPES buffer for immunoprecipitation assays, and platelet aggregation was performed as described above. The reaction was terminated using an equal volume of ice-cold NP40 lysis buffer (2% [v/v] Nonidet™ P-40, 50 mM Tris, 20 mM NaF, 25 mM *β*-glycerophosphate, 120 mM NaCl, 10 mM ethylenediaminetetraacetic acid [EDTA], 1 mM phenylmethylsulfonyl fluoride [PMSF], 2 mM Na_3_VO_4_, 5 mg/mL leupeptin, 5 mg/mL aprotinin, 1 mg/mL pepstatin A, and 1 mM benzamide, pH 7.5). The lysate obtained was precleared with protein A-sepharose (PAS) for 1 h at 4°C. PAS was then removed and the lysate treated with pull-down antibody overnight at 4°C on a rotary shaker. Protein/antibody complexes were isolated with PAS for 4 h at 4°C and washed five times with lysis buffer. The beads were boiled in Laemmli sample buffer (2% sodium dodecyl sulfate [SDS], 1%  *β*-mercaptoethanol, 0.008% bromophenol blue, 80 mM Tris/HCl pH 6.8, and 1 mM EDTA), and the proteins resolved using SDS-polyacrylamide gel electrophoresis (SDS-PAGE). The resolved proteins were transferred to polyvinylidene difluoride (PVDF) membranes; the membranes were blocked with 5% milk, probed with respective primary and secondary antibodies, and visualized using enhanced chemiluminescence (Advansta, CA, USA).

### 2.10. Immunoblotting

Platelet aggregation was terminated by the addition of lysis buffer (PRO-PREP; iNtRON Biotechnology, Seoul, Korea) to the mixture, followed by the estimation of protein concentration using the BCS assay (PRO-MEASURE; iNtRON Biotechnology, Seoul, Korea). Total platelet proteins were separated using 10% SDS-PAGE and transferred to PVDF membranes. Membranes were blocked with 5% skim milk, probed with respective antibodies, and visualized using enhanced chemiluminescence (Advansta, CA, USA).

### 2.11. Statistical Analysis

Data were analyzed by one-way analysis of variance (ANOVA), followed by Dunnett's post hoc tests to measure statistical significance among and between measurements (SAS Institute Inc., Cary, NC, USA). All data are presented as the means ± standard errors of the mean (SEMs). A* p* value of 0.05 or less was considered statistically significant.

## 3. Results

### 3.1. A. chinensis Inhibits Collagen-Induced Platelet Aggregation

**Figure 1:**
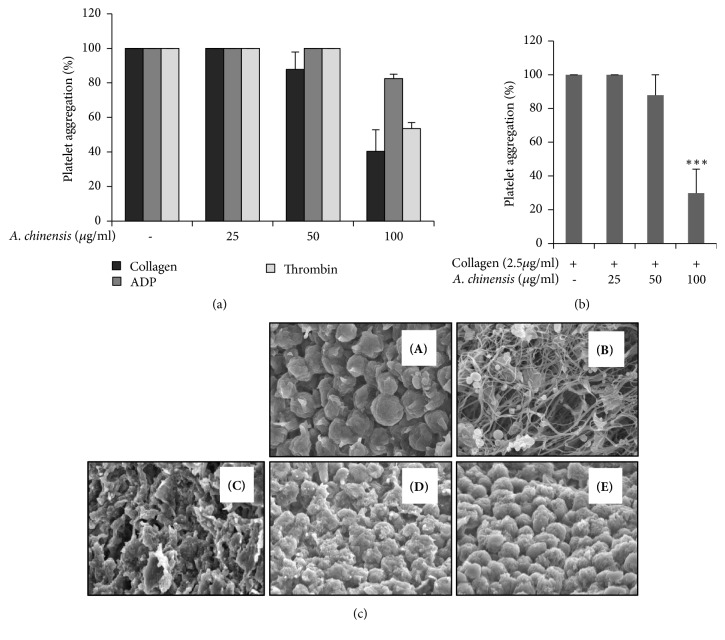
*Astilbe chinensis* extract inhibits collagen-stimulated platelet aggregation. (a–c) Washed platelets were pretreated with* A. chinensis* extract or vehicle for 2 min in the presence of 1 mM CaCl_2_ and then stimulated with collagen for 5 min. (c) After the platelet aggregation reaction, scanning electron microscopy was performed. Representative scanning electron microscopy images of collagen (2.5 *μ*g/mL)-stimulated platelets pretreated with various concentrations of* A. chinensis* extract [(A) Resting, (B) Vehicle, (C) 25 *μ*g/mL, (D) 50 *μ*g/mL, and (E) 100 *μ*g/mL]. The graph represents the means ± SEMs of at least four independent experiments. *∗∗∗p*< 0.001 versus control.

Initial screening with different agonists showed that* A. chinensis* extract inhibits collagen-induced platelet aggregation (Figure 1(a)). A significant and concentration-dependent inhibition of platelet aggregation was observed in platelets pretreated with* A. chinensis *extract compared with that of vehicle control (Figure 1(b)). Activation of platelet receptors (e.g., GPVI or P2Y12) triggers downstream signaling events which causes granule secretion, platelet shape change, and fibrin formation, subsequently leading to platelet aggregation. The continuous change in shape from the unactivated to the fully activated platelet is best seen on scanning electron microscopy. We confirmed these results using scanning electron microscopy to show inhibition of platelet shape change and platelet aggregation. A clear inhibition in platelet shape change can be seen in collagen-stimulated platelets pretreated with increasing concentrations of* A. chinensis* as compared with vehicle stimulated platelets (Figure 1(c)).

### 3.2. A. chinensis Reduces [Ca^*2*+^]_i_ Mobilization and ATP Secretion

**Figure 2:**
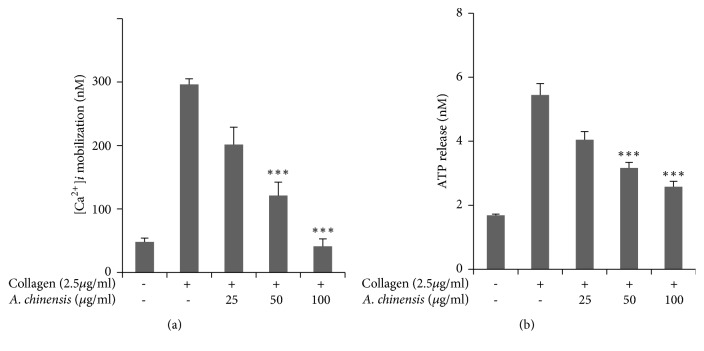
The inhibitory effect of* Astilbe chinensis* on collagen-stimulated [Ca^2+^]_*i*_ elevation and ATP secretion. (a) Washed platelets were loaded with a calcium fluorophore (5 *μ*M, Fura-2/AM) for 1 h. Fura 2/AM-loaded platelets were pretreated with* A. chinensis* for 2 min at 37°C and stimulated with collagen. (b) After platelet aggregation was terminated, the concentration of ATP was assessed in collagen-stimulated platelets treated with* A. chinensis* extract, using a luminometer. The results represent the mean ± SEM of at least four independent experiments. *∗∗∗p*< 0.001 versus control.

Calcium mobilization was assessed in platelets pretreated with* A. chinensis* extract or vehicle, and the results showed significant, concentration-dependent inhibition in intracellular calcium elevation, which indicates that* A. chinensis* extract may inhibit platelet aggregation by inhibiting calcium mobilization (Figure 2(a)). Granule secretion plays an important role in platelet activation and enhances platelet aggregation. ATP release was assessed in collagen-stimulated platelets pretreated with* A. chinensis* extract or vehicle.* A. chinensis *extract was found to inhibit ATP secretion in a significant manner (Figure 2(b)).

### 3.3. A. chinensis Inhibits Fibrinogen Binding to Integrin *α*_IIb_*β*_*3*_

**Figure 3:**
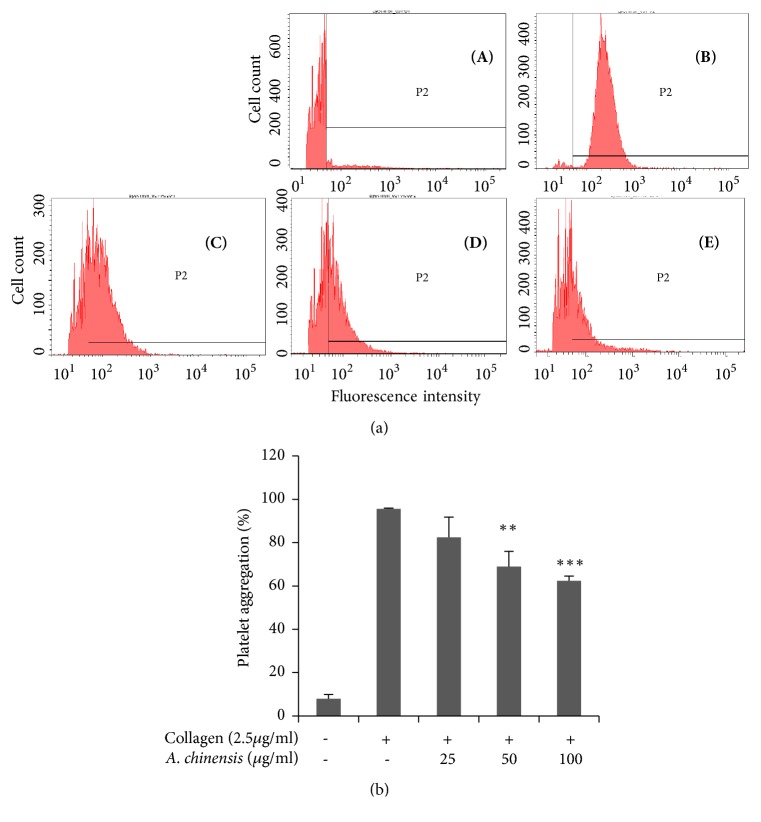
*Astilbe chinensis* extract blocks fibrinogen binding to integrin *α*_IIb_*β*_3_ in collagen-induced platelets. (a) Flow cytometry was used to measure fibrinogen binding to collagen (2.5 *μ*g/mL)-stimulated platelets pretreated with various concentrations of* A. chinensis* extract [(A) Resting, (B) Vehicle, (C) 25 *μ*g/mL, (D) 50 *μ*g/mL, and (E) 100 *μ*g/mL]. (b) Bar graph summarizing the inhibitory effect of* A. chinensis* extract on fibrinogen binding to integrin *α*_IIb_*β*_3_. *∗∗* p < 0.01 and *∗∗∗p*< 0.001 versus control.

Fibrinogen binding to integrin *α*_IIb_*β*_3_ causes a conformational change in integrin *α*_IIb_*β*_3_ structure, which is also known as inside-out signaling, consequently leading to platelet aggregation. Our extract showed significant and dose dependent inhibition of fibrinogen binding to integrin *α*_IIb_*β*_3_ (Figure 3).

### 3.4. A. chinensis Reduces Phosphorylation of MAPK and MEK Signaling Molecules and PI3K/Akt Activation

**Figure 4:**
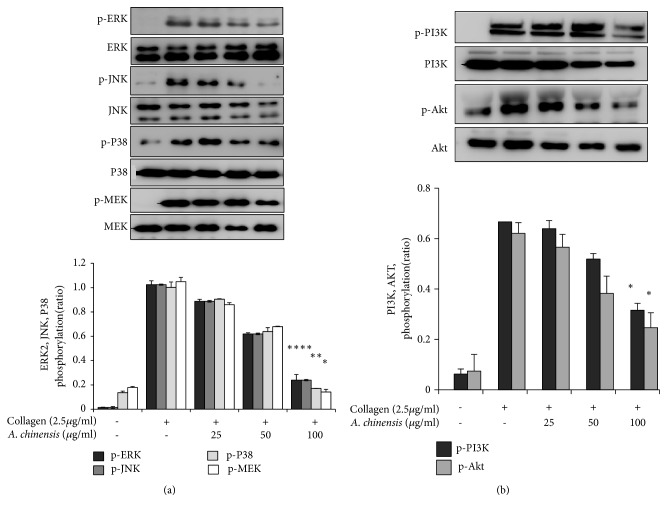
*Astilbe chinensis* extract attenuates mitogen-activated protein kinases (MAPKs) and MEK (a), and phosphoinositide 3-kinase (PI3K)/Akt (b) phosphorylation in collagen-stimulated platelets. Washed platelets were pretreated with* A. chinensis* extract and stimulated with collagen. After the reaction was terminated, protein was extracted from the platelets and analyzed for the phosphorylation of the indicated proteins by immunoblot analysis. Representative immunoblot images are shown, with the quantified data plotted below (mean ± SEM, n = 3). *∗p*< 0.05 and *∗∗p*< 0.01 versus the agonist-treated group.

Previous studies have shown that the MAPKs, ERK, JNK, and p38 are highly expressed in agonist-induced activated platelets and play vital roles in platelet activation [16].* A. chinensis* extract significantly inhibited phosphorylation of all MAPK molecules in a concentration-dependent manner (Figure 4(a)). MEK is an upstream molecule in the ERK pathway [17], and* A. chinensis* extract significantly and concentration-dependently inhibited phosphorylation of this protein (Figure 4(a)).

The PI3K/Akt pathway has been shown to play an important role in platelet aggregation [18]. Therefore, we assessed activation of this pathway in collagen-stimulated platelets pretreated with* A. chinensis *extract and found that our extract had significant inhibitory potential against the activation of this pathway (Figure 4(b)). These results show that* A. chinensis *extract potentially inhibits platelet aggregation by inhibiting phosphorylation of MAPK and MAPKK signaling proteins and PI3K/Akt pathway activation.

### 3.5. A. chinensis Inhibits Phosphorylation of Proteins Involved in GPVI Signaling

**Figure 5:**
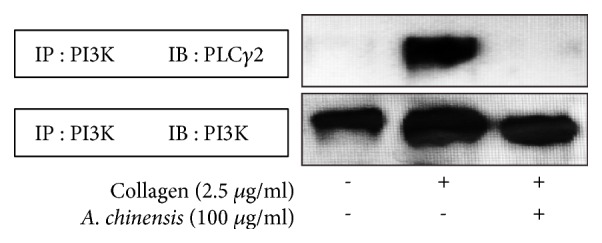
*Astilbe chinensis* extract attenuates collagen-stimulated phospholipase C*γ*2 (PLC*γ*2) activation in the glycoprotein VI (GPVI) signaling pathway. Washed platelets were preincubated with* A. chinensis *extract or vehicle for 3 min and then were stimulated with collagen for 5 min. Lysates (100 *μ*g/mL) from* A. chinensis* extract-treated platelets were immunoprecipitated by incubating overnight with anti-PI3K antibody and then incubating with protein A-sepharose for 4 h at 4°C. Immunoprecipitated proteins were separated by SDS-PAGE and immunoblotted to detect PLC*γ*2 expression.

The proteins P13K and phospholipase C*γ*2 (PLC*γ*2) play significant roles in GPVI signaling and are crucial contributors to downstream outcomes, such as granule secretion and platelet aggregation [19]. Therefore, we examined the effect of* A. chinensis* extract on these proteins in the GPVI pathway. Our results show that* A. chinensis *extract markedly blocked interactions between the two proteins (Figure 5).

## 4. Discussion

Platelets are small fragments derived from megakaryocytes that help to maintain hemostasis and prevent blood loss by inducing fibrin clot formation at the site of vascular injury. However, hyperactivation of platelets may prove fatal and contributes to the formation of atherosclerotic plaques within blood vessels, which progressively restricts blood flow, leading to hypoxia and ischemic injury. Plaques can also rupture from vessels making emboli, leading to stroke and myocardial infarction [20]. A number of antiplatelet drugs are available and have proven to be beneficial in reducing the risk of thrombotic events. However, these drugs can produce undesirable side effects and complications, and they are ineffective in some patients [21], which necessitates the development of safer approaches to treat CVDs. Natural compounds have been shown to reduce CVDs [8], and, in our effort to discover alternative compounds, we found* A. chinensis*. These medicinal plants have been used in China, Korea, and Japan in traditional remedies [11, 22].

In the present study, we evaluated the inhibitory effects of* A. chinensis* extract on platelet aggregation and explored the mechanistic aspects of these extracts on platelet function. These extracts regulated [Ca^2+^]_*i*_ mobilization and inhibited ATP secretion. Moreover, fibrinogen binding to integrin *α*_IIb_*β*_3_ was inhibited, and activation of the MAPK and PI3k/Akt pathways was attenuated. Our results show that* A. chinensis* extract significantly inhibited [Ca^2+^]_*i*_ mobilization in a concentration-dependent manner, and dense granule secretion. Intracellular calcium plays a critical role in platelet activation, aggregation, and thrombus formation [23]. Increased [Ca^2+^]_*i*_ triggers granule, which enhances platelet activation, while calcium chelation inhibits ATP release (dense granule secretion) [24]. Comparing our results with those in the literature, our data suggest that* A. chinensis* extract exerts inhibitory effects on granule secretions to regulate platelet function.

Activation of platelets leads to conformational changes in the structure of integrin *α*_IIb_*β*_3_, which enhances platelet aggregation [25]. Therefore, integrin inactivation is of great interest in the development of antiplatelet therapy. Altered fibrinogen binding to integrin *α*_IIb_*β*_3_ in response to these conformational changes is also known as inside-out signaling, and later steps involve further signal transduction that is necessary for complete platelet aggregation [26]. Our results show that fibrinogen binding to integrin *α*_IIb_*β*_3_ was significantly inhibited by pretreatment of platelets with* A. chinensis* extract, which indicates that pretreatment of platelets with these extracts may attenuate the conformational changes (i.e., inside-out signaling) and impair integrin *α*_IIb_*β*_3_ activation.

Many studies have shown that platelets are continuously exposed to several factors that cause their activation and aggregation, such as collagen, ADP, thrombin, fibrinogen, von Willebrand factor (vWF), and thromboxane; some factors are also inhibitory, such as prostacyclin (PGI_2_) and ADPase [27]. Any imbalance in these opposing factors may cause impairment in hemostasis; thus, a strong equilibrium is necessary for normal platelet function. Our results indicate that pretreatment of platelets with* A. chinensis* extract may contribute to maintaining this balance and hemostasis.

Platelets express MAPK that include ERK, JNK, and p38^MAPK^, which are activated by several agonists (e.g., collagen, ADP, and thrombin) [16]. ERK2 has been shown to enhance collagen-stimulated platelet secretion, while p38 is involved in platelet spreading and adhesion. ERK2 and p38 inhibitors have been shown to suppress platelet activation [28]. PI3K and PLC*γ*2 play important roles in collagen-stimulated GPVI downstream signaling and are involved in platelet granule secretion and aggregation [29]. Previous studies have shown that the PI3K/Akt pathway is involved in cardiac protection by inducing antiapoptotic effects and reducing myocardial ischemia reperfusion injury (MI/RI) [30]. Moreover, studies have shown that platelet aggregation can be reversed in collagen- or ADP-stimulated platelets by blocking PI3K [31].

Our results show that* A. chinensis* extract inhibited the phosphorylation of these molecules, indicating that MAPK proteins and the PI3K/Akt pathway may be involved in the antiplatelet mechanism of* A. chinensis* extract. Further,* A. chinensis* extracts inhibited PI3K and PLC*γ*2 interaction, indicating their possible mechanism of action in inactivating GPVI receptor downstream signaling. Moreover, our findings suggest that* A. chinensis *extract inhibits PI3K/Akt signaling and its involvement in modulating the reperfusion injury salvage kinase (RISK) pathway.

## 5. Conclusion

Our results show that* A. chinensis* extract is potent inhibitor of collagen-induced platelet aggregation, granule secretion, and fibrinogen binding to integrin *α*_IIb_*β*_3_. Moreover, the extract modulates platelet function* via* impaired MAPK phosphorylation and inactivation of PI3K/Akt pathway, suggesting their antiplatelet potential. We suggest that* A. chinensis* extract is potent antithrombotic candidate that can be used to treat platelet-related cardiovascular disorders.

## Data Availability

All data generated or analyzed during this study are included in this published article.
